# Supplementary material for: Natural statistics support a rational account of confidence biases
Source: Nat Commun. 2023 Jul 6;14:3992. doi: 10.1038/s41467-023-39737-2 (PMC10326055; doi:10.1038/s41467-023-39737-2)
Supplement: Supplementary file 1 — Supplementary information [file 41467_2023_39737_MOESM1_ESM.pdf]

Supplementary Figures

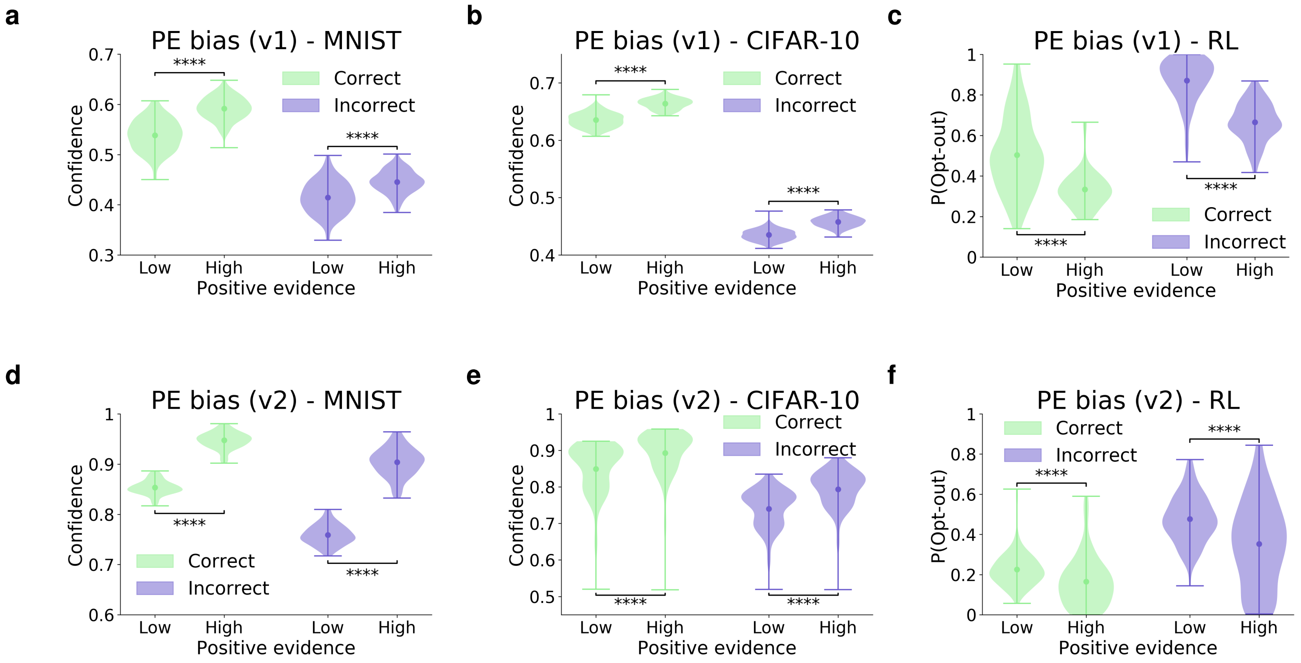

Figure S1: **Confidence for both correct and incorrect trials shows a positive evidence bias.** Panels **(a)** (MNIST), **(b)** (CIFAR-10), and **(c)** (RL orientation discrimination task), show confidence for correct and incorrect and trials, for the version of the PE bias involving manipulation of contrast and noise (two-sided paired t-tests; correct trials: MNIST, $p = 6.8 \times 10^{-27}$; CIFAR-10, $p = 3.2 \times 10^{-54}$; RL, $p = 1.5 \times 10^{-18}$; incorrect trials: MNIST, $p = 5.2 \times 10^{-13}$; CIFAR-10, $p = 3 \times 10^{-46}$; RL, $p = 4.5 \times 10^{-35}$). Panels **(d)** (MNIST), **(e)** (CIFAR-10), and **(f)** (RL orientation discrimination task), show confidence for correct and incorrect and trials, for the version of the PE bias involving superimposed stimuli presented at different contrast levels (two-sided paired t-tests; correct trials: MNIST, $p = 4.3 \times 10^{-83}$; CIFAR-10, $p = 3.6 \times 10^{-45}$; RL, $p = 1.2 \times 10^{-9}$; incorrect trials: MNIST, $p = 2.9 \times 10^{-79}$; CIFAR-10, $p = 1.1 \times 10^{-50}$; RL, $p = 1 \times 10^{-10}$). In all cases, confidence shows a significant PE bias for both correct and incorrect trials. All results reflect the probability density over 100 trained networks, with mean confidence in each condition represented by circular markers, and maxima/minima represented by the upper/lower lines; **** indicates $p < 0.0001$. Source data are provided as a Source Data file.

	Standard	Fixed μ/σ	Fixed μ	Fixed σ
Standard vs.		$p = 2.5 \times 10^{-5}$	$p = 0.004$	$p = 2 \times 10^{-6}$
Fixed μ/σ vs.	$p = 1.4 \times 10^{-8}$		$p = 6.9 \times 10^{-12}$	$p = 6.8 \times 10^{-32}$
Fixed μ vs.	$p = 0.02$	$p = 0.003$		$p = 1.4 \times 10^{-13}$
Fixed σ vs.	$p = 0.68$	$p = 4.1 \times 10^{-6}$	$p = 0.006$	

Table S1: **Statistical comparison of meta-d' for four test regimes.** For each test regime (rows), 200 networks trained on that regime (e.g., networks trained and tested on the standard regime) are compared (two-sided two-sample t-tests) to 200 networks trained in each of the other three regimes (e.g., networks trained on the fixed μ regime and tested on the standard regime). Each cell represents the results of a two-sample t-test.

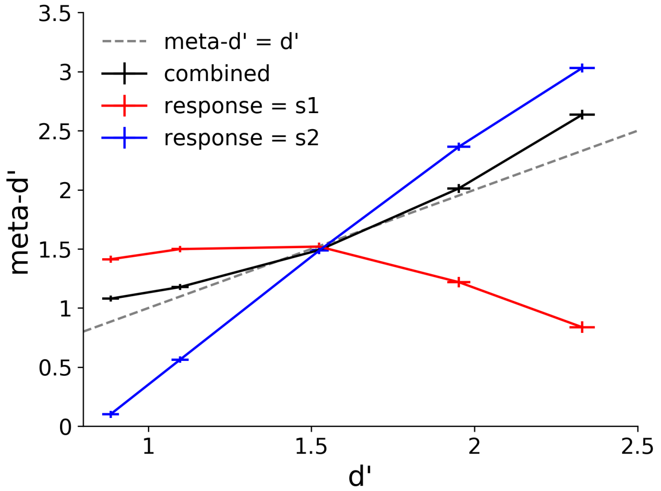

Figure S2: **Dissociation between type-1 and type-2 sensitivity is present without additional type-2 noise.** With no additional type-2 noise ($\xi = 0$), overall meta-d' is higher than in the original behavioral results, but the major qualitative effects are still present. The meta-d' vs. d' function has a negative slope for trials with a response of s1, and a positive slope for trials with a response of s2. Source data are provided as a Source Data file.

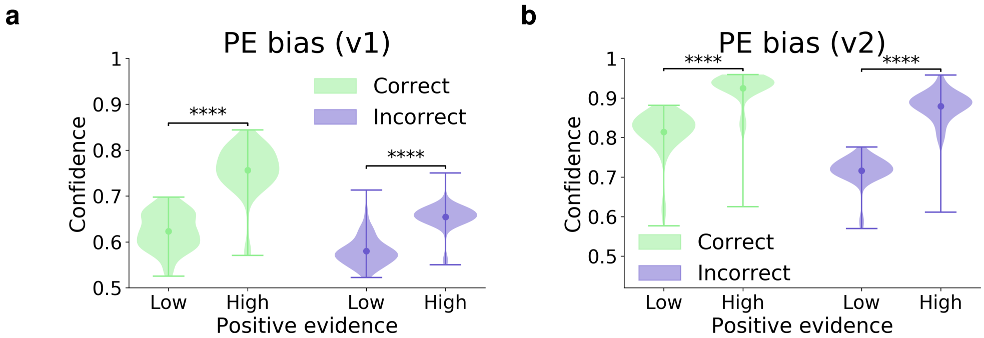

Figure S3: **Ideal observer shows PE bias for both correct and incorrect trials.** Ideal observer confidence for correct and incorrect trials, for **(a)** version of PE bias involving manipulation of contrast and noise (two-sided paired t-tests; correct trials: $p = 5.3 \times 10^{-65}$; incorrect trials: $p = 2.7 \times 10^{-46}$), and **(b)** version of the PE bias involving superimposed stimuli presented at different contrast levels (two-sided paired t-tests; correct trials: $p = 2.1 \times 10^{-57}$; incorrect trials: $p = 6.1 \times 10^{-76}$). All results reflect the probability density over 100 trained networks, with mean confidence in each condition represented by circular markers, and maxima/minima represented by the upper/lower lines; **** indicates $p < 0.0001$. Source data are provided as a Source Data file.

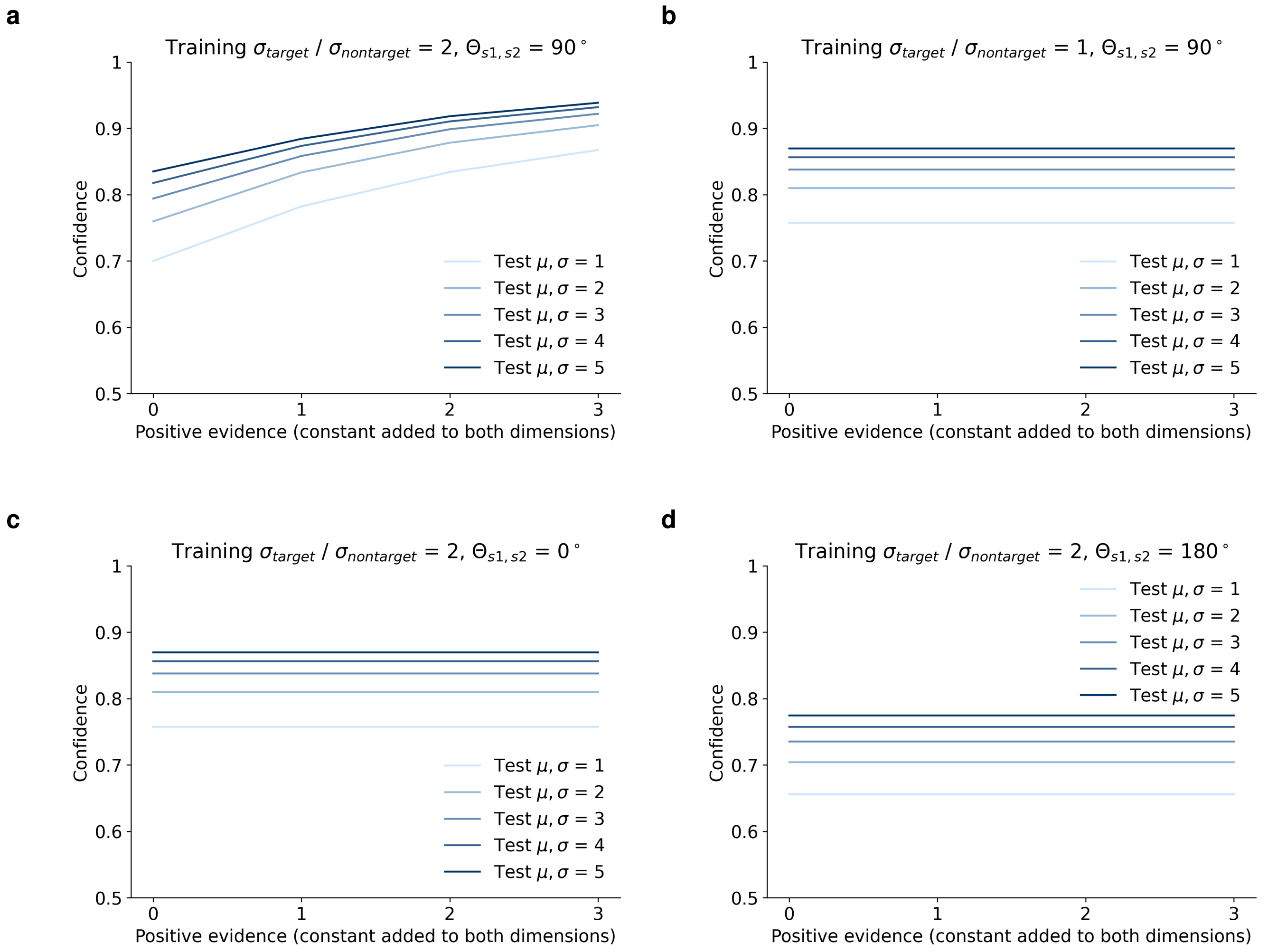

Figure S4: **Dependence of PE bias on sensory evidence variance structure.** Analysis of both versions of PE bias as a function of the variance structure of the sensory evidence distributions. Ideal observer was implemented for training distributions that varied in terms of two properties: 1) $\sigma_{target}/\sigma_{nontarget}$, the ratio of the variance in the target vs. nontarget dimensions (intuitively, the extent to which the training distributions had an elliptical shape), and 2) $\theta_{s1,s2}$, the angular difference between the major axes of the distributions for stimulus classes s1 and s2. Ideal observer was evaluated on test distributions with equal variance in the target and nontarget dimensions (i.e., circular distributions), with a signal-to-noise ratio corresponding to an accuracy of 75% for all conditions. Colored lines correspond to distributions that varied in both their target mean μ (the mean in the dimension corresponding the correct answer) and their variance σ. Thus, version 1 of the PE bias corresponds to an increase in confidence between the light blue and dark blue lines. This version of the PE bias emerged under all variance conditions. The X axes represent the value of a constant added to both dimensions. Thus, version 2 of the PE bias corresponds to an increase in confidence as a function of the X axis. **(a)** This version of the PE bias only emerged when sensory evidence distributions were asymmetric ($\sigma_{target}/\sigma_{nontarget} > 1$) and non-parallel ($0^\circ < \theta_{s1,s2} < 180^\circ$). When sensory evidence distributions had **(b)** equal variance in both dimensions ($\sigma_{target}/\sigma_{nontarget} = 1$), or had major axes with an angular difference of either **(c)** $\theta_{s1,s2} = 0^\circ$ or **(d)** $\theta_{s1,s2} = 180^\circ$, the ideal observer did not show this version of the PE bias. Source data are provided as a Source Data file.

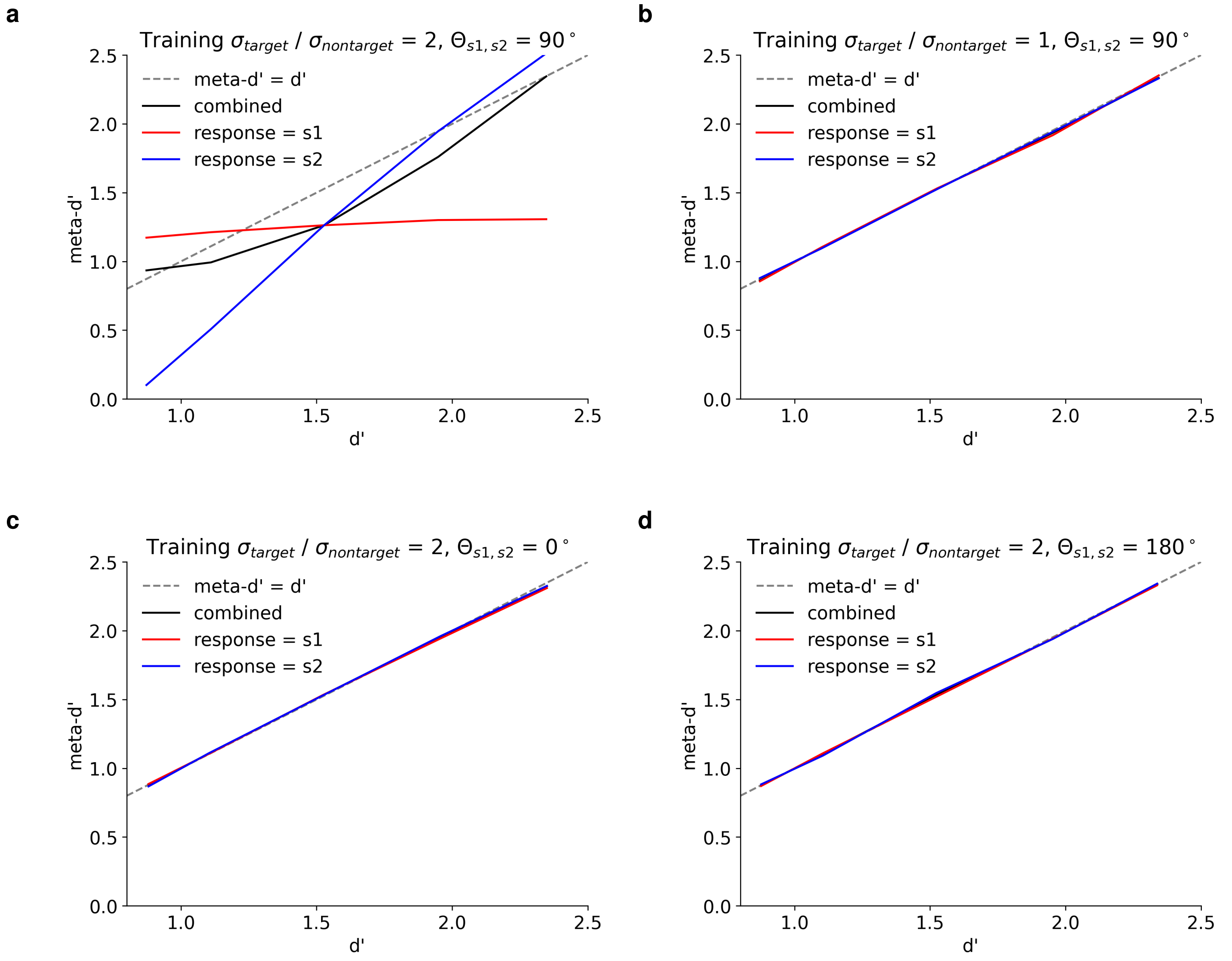

Figure S5: **Dependence of dissociation between type-1 and type-2 sensitivity on sensory evidence variance structure.** **(a)** Dissociation between type-1 and type-2 sensitivity emerged only when sensory evidence distributions were asymmetric ($\sigma_{target}/\sigma_{nontarget} > 1$) and non-parallel ($0° < \theta_{s1,s2} < 180°$). When sensory evidence distributions had **(b)** equal variance in both dimensions ($\sigma_{target}/\sigma_{nontarget} = 1$), or had major axes with an angular difference of either **(c)** $\theta_{s1,s2} = 0°$ or **(d)** $\theta_{s1,s2} = 180°$, the ideal observer did not show this dissociation. Source data are provided as a Source Data file.

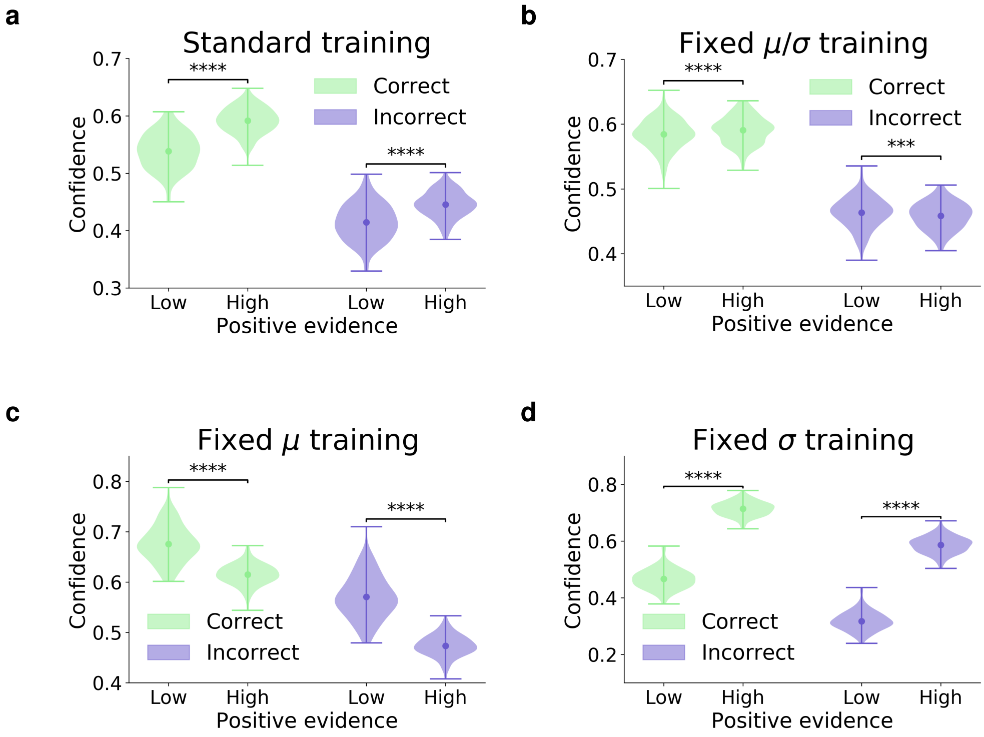

Figure S6: **Confidence for correct and incorrect trials under alternative training regimes.** When trained on the **(a)** standard (two-sided paired t-tests; correct trials: $p = 6.8 \times 10^{-27}$; incorrect trials: $p = 5.2 \times 10^{-13}$) or **(d)** fixed σ regimes (two-sided paired t-tests; correct trials: $p = 6.7 \times 10^{-76}$; incorrect trials: $p = 4.6 \times 10^{-79}$), the model showed a PE bias for both correct and incorrect trials. When trained on the **(c)** fixed μ regime, the model showed a reversed PE bias for both correct and incorrect trials (two-sided paired t-tests; correct trials: $p = 1.2 \times 10^{-29}$; incorrect trials: $p = 3.5 \times 10^{-40}$). When trained on the **(b)** fixed μ/σ regime, the model showed a very small PE bias for correct trials, and a very small reversed PE bias for incorrect trials (two-sided paired t-tests; correct trials: $p = 3.3 \times 10^{-6}$; incorrect trials: $p = 0.0003$). These effects canceled each other out, such that there was no overall PE bias for models trained on this regime. All results reflect the probability density over 100 trained networks, with mean confidence in each condition represented by circular markers, and maxima/minima represented by the upper/lower lines; *** indicates $p < 0.001$, **** indicates $p < 0.0001$. Source data are provided as a Source Data file.

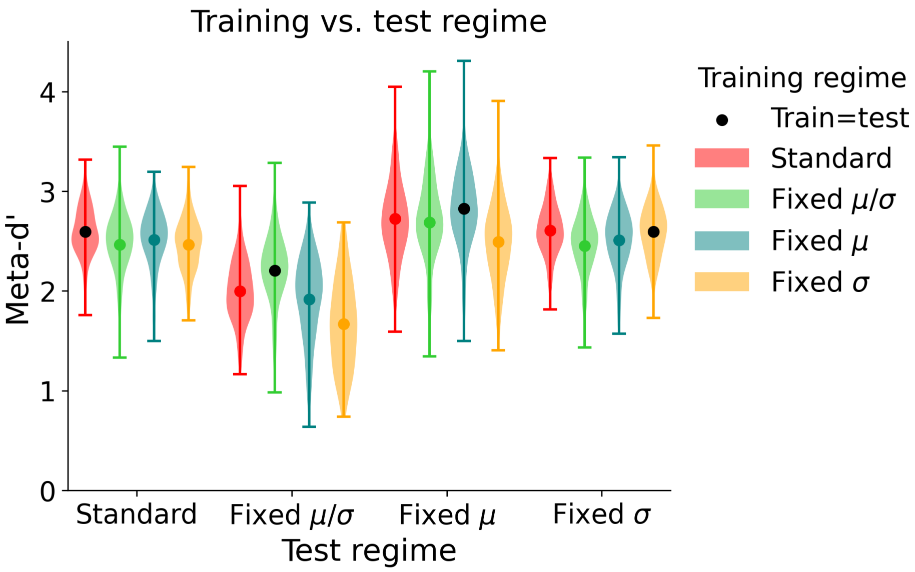

Figure S7: **Metacognitive sensitivity under alternative training regimes.** Metacognitive sensitivity (as measured by meta-d') in each test regime as a function of a network's training regime. In general, for each test regime, the best metacognitive performance was achieved by networks trained in that regime (as indicated by black markers; note that distinct training and test images were used even when the training and test regime were the same). The one exception is the fixed σ test regime, on which networks trained in both the standard and fixed σ regime – both of which displayed a PE bias – performed equally well. Results reflect the probability density over 200 trained networks, with mean meta-d' in each condition represented by circular markers, and maxima/minima represented by the upper/lower lines; statistical comparisons are omitted for visual clarity, but are presented in Supplementary Table S1. Source data are provided as a Source Data file.

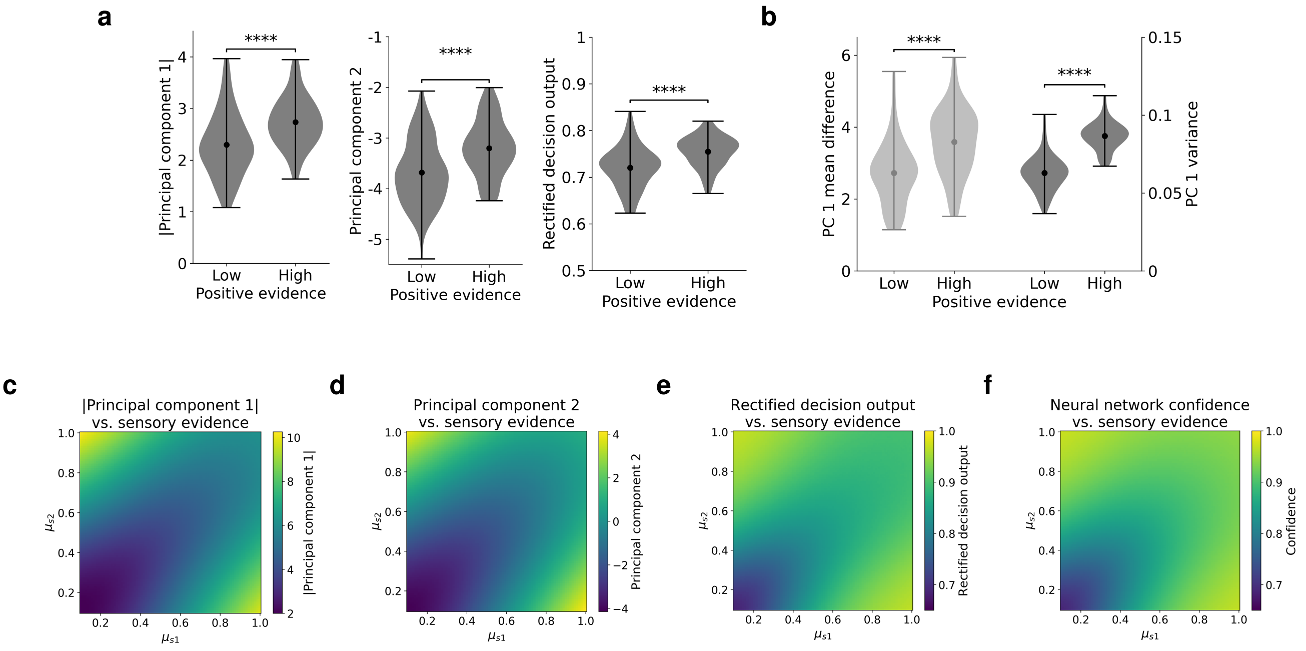

Figure S8: **Common decision variable is subject to same biases as confidence.** **(a)** The common decision variable, used for both decisions and confidence, is subject to the PE bias (version 1), as seen in the rectified values along PC1 (|PC1|; two-sided paired t-test, $p = 1.8\times10^{-20}$), PC2 (two-sided paired t-test, $p = 6.4\times10^{-15}$), and the rectified values of the network's decision output (the probability that the network assigns to the chosen decision; two-sided paired t-test, $p = 4 \times 10^{-19}$), but cannot be seen in decisions themselves, which only take into account the most likely choice. **(b)** The means of the distributions for s1 and s2 along PC1 are more separated in the high vs. low PE conditions (two-sided paired t-test, $p = 5.8 \times 10^{-27}$), but these distributions also have greater variance in the high PE condition (two-sided paired t-test, $p = 6.4 \times 10^{-40}$), explaining the balanced accuracy across these conditions. Panels **(b)** and **(c)** reflect the probability density over 100 trained networks, with the mean in each condition represented by circular markers, and maxima/minima represented by the upper/lower lines; **** indicates $p < 0.0001$. **(c)**-**(f)** Visualizing the decision variable as a function of the sensory evidence in favor of each stimulus class. **(c)** |PC1| displays a nearly identical pattern to **(d)** PC2. **(e)** Rectified decision output displays a nearly identical pattern to **(f)** confidence. Note that the rectified decision output and confidence differ from |PC1| and PC2 due to the addition of a sigmoid nonlinearity (see Methods Section 4.3.2). Panels **(c)**-**(f)** reflect an average over 100 trained networks. Source data are provided as a Source Data file.

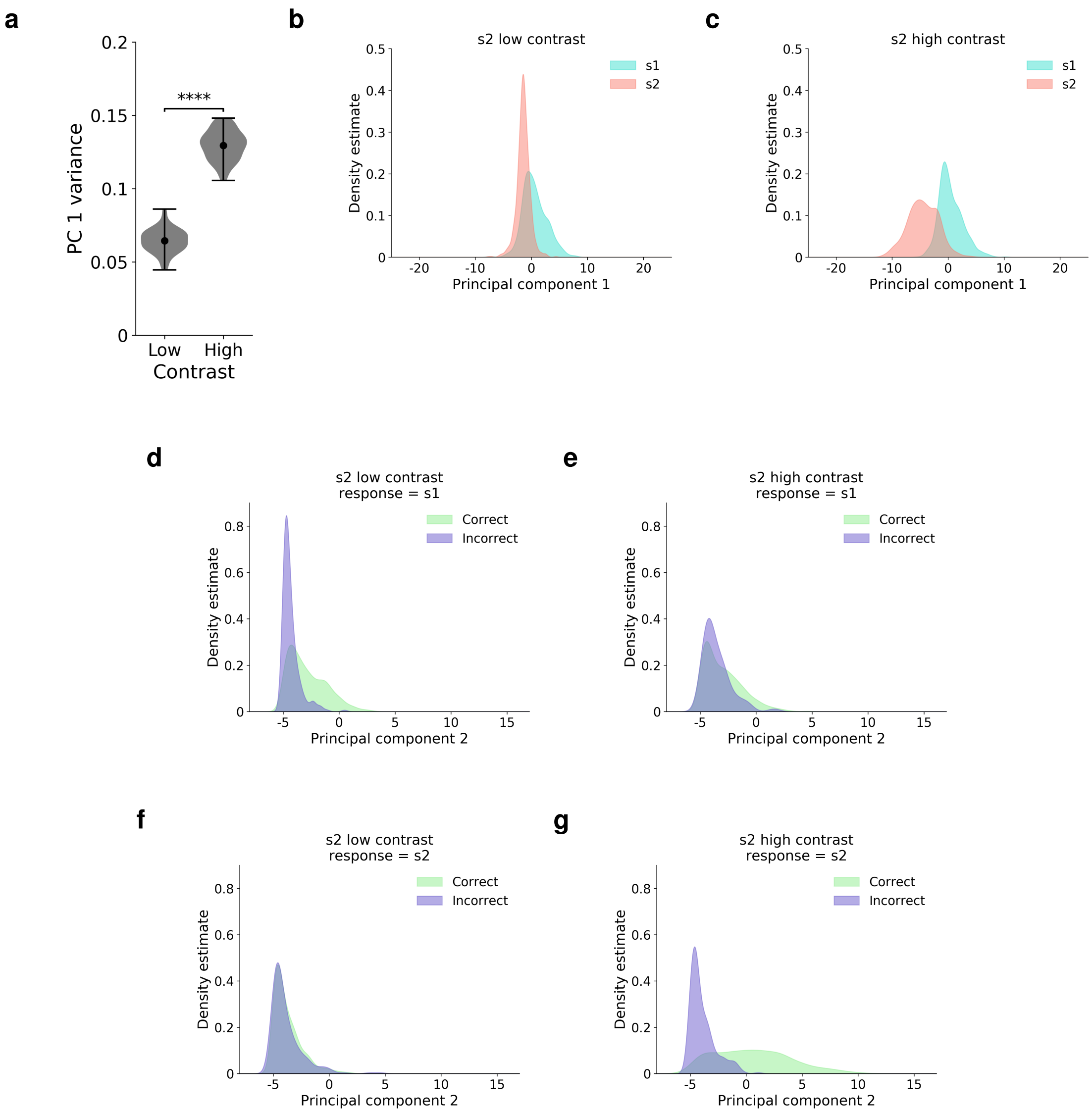

Figure S9: **Role of learned low-dimensional representations in dissociation between type-1 and type-2 sensitivity.** **(a)** Stimulus distributions along PC1 have greater variance for high vs. low contrast images (two-sided paired t-test, $p = 1.6 \times 10^{-70}$). This is the case even when the same noise level is used for both conditions. Results reflect the probability density over 100 trained networks, with the mean in each condition represented by circular markers, and maxima/minima represented by the upper/lower lines; **** indicates $p < 0.0001$. Networks were trained on the two-choice variant of MNIST. For evaluation, the noise level was set to a value of $\sigma = 2$, and images were presented at a contrast of either $\mu = 0.1$ or $\mu = 1$. This property helps to explain the observed dissociation between type-1 and type-2 sensitivity. **(b)** Results from an example network, trained on the two-choice variant of MNIST, and evaluated on the task depicted in Figure 3e. When s2 is presented at a lower contrast than s1, the distribution for s2 has lower variance than the distribution for s1. **(c)** When s2 is presented at a higher contrast than s1, the distribution for s2 has higher variance than the distribution for s1. This means that, for trials with a response of s1 (PC1 $>$ 0 in this example), distributions along PC2 for correct vs. incorrect trials have greater overlap (resulting in lower meta-d') when s2 is presented at high **(e)** vs. low **(d)** contrast. For trials with a response of s2 (PC1 $<$ 0 in this example), correct vs. incorrect trials have substantially less overlap (resulting in higher meta-d') when s2 is presented at high **(g)** vs. low **(f)** contrast. The crossover pattern exhibited by meta-d' in Figure 3f can therefore be explained as an emergent consequence of the positive correlation between signal strength and variability that characterizes the model's learned low-dimensional representations. Note that a nearly identical pattern is seen when looking at $|$PC1$|$ instead of PC2 (Figure S10), confirming that this dissociation can be explained by a model that utilizes a common decision variable for both decisions and confidence. Source data are provided as a Source Data file.

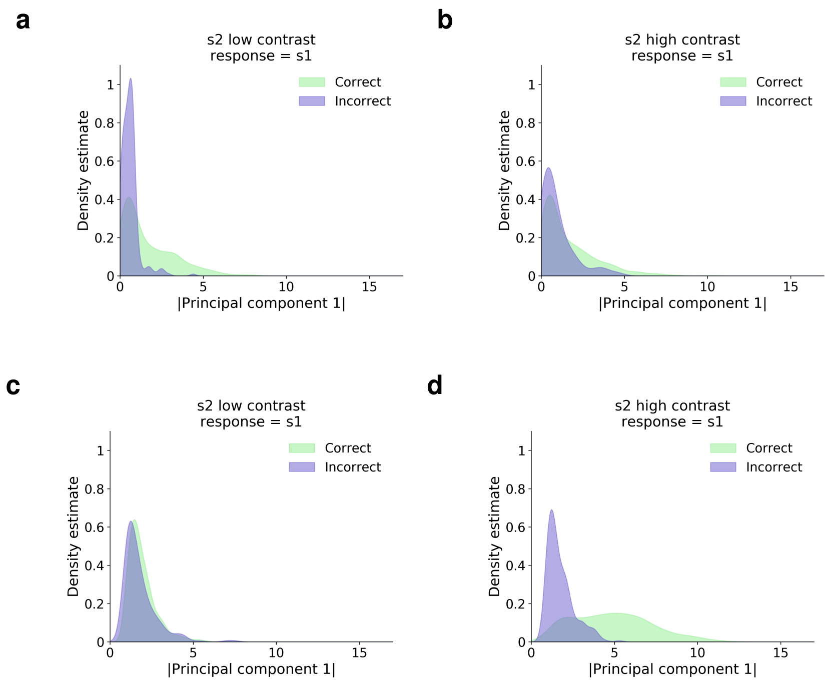

Figure S10: **Role of learned low-dimensional representations in dissociation between type-1 and type-2 sensitivity (continued from Figure S9).** Panels **(a)-(d)** depict results of the same analysis as Figures S9d- S9g, but applied to |PC1| instead of PC2. This analysis yields qualitatively similar results for both |PC1| and PC2, supporting an explanation of the dissociation between type-1 and type-2 sensitivity in terms of a single decision variable. See caption of Figure S9 for further explanation. Source data are provided as a Source Data file.

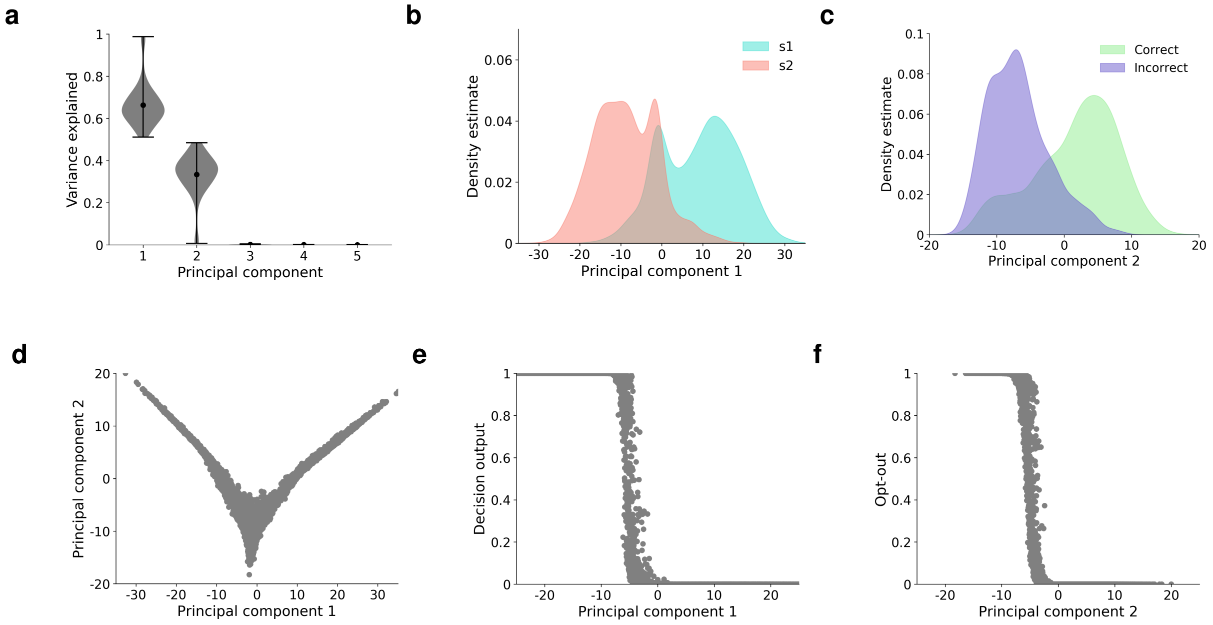

Figure S11: **Learned representations extract a common decision variable in RL task.** **(a)** Learned representations in penultimate layer were almost entirely explained by the top two principal components. Panel **(a)** reflects the probability density over 100 trained networks, with the mean for each component represented by circular markers, and maxima/minima represented by the upper/lower lines. Panels **(b)**-**(f)** depict results from a single trained network for the purposes of illustrating the model's learned two-dimensional geometry, but this general representational scheme was shared by all networks. **(b)** Kernel density estimates show that the distributions for s1 vs. s2 were separated along PC1. **(c)** The distributions for correct vs. incorrect trials were separated along PC2. **(d)** PC2 closely resembled a rectification of PC1. **(e)** PC1 predicted the model's decisions. **(f)** PC2 predicted the strength of the model's opt-out response. Panels **(d)**-**(f)** depict trial-by-trial data points, not the predictions of a regression model. Source data are provided as a Source Data file.

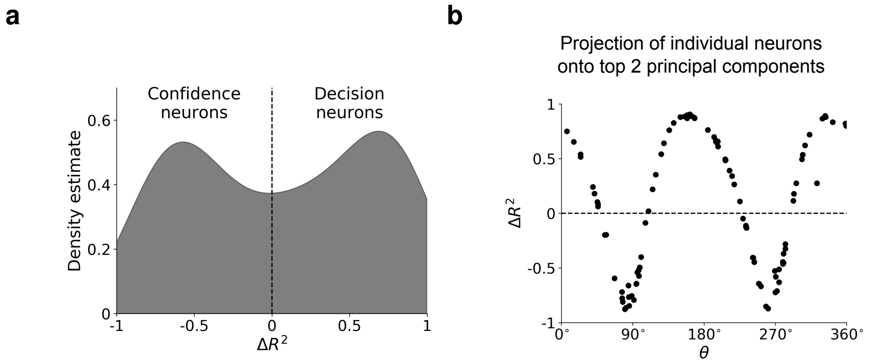

Figure S12: **Additional analysis of single unit representations.** **(a)** Confidence vs. decision neurons were classified by computing $\Delta R^2 = R^2_{decision} - R^2_{opt-out}$, with a criterion at $\Delta R^2 = 0$. **(b)** ΔR^2 was strongly predicted by the projection of each neuron onto the top 2 PCs. Neurons aligned with PC1 ($\theta \approx 0^\circ$ or $\theta \approx 180^\circ$) were strongly predictive of decisions ($\Delta R^2 \to 1$), while neurons aligned with PC2 ($\theta \approx 90^\circ$ or $\theta \approx 270^\circ$) were strongly predictive of confidence ($\Delta R^2 \to -1$). Other neurons were moderately predictive of both decisions and confidence (resulting in lower ΔR^2), and were not strongly aligned with either of the top 2 PCs. Results reflect a single example network, but all 100 trained networks showed a similar pattern. Source data are provided as a Source Data file.

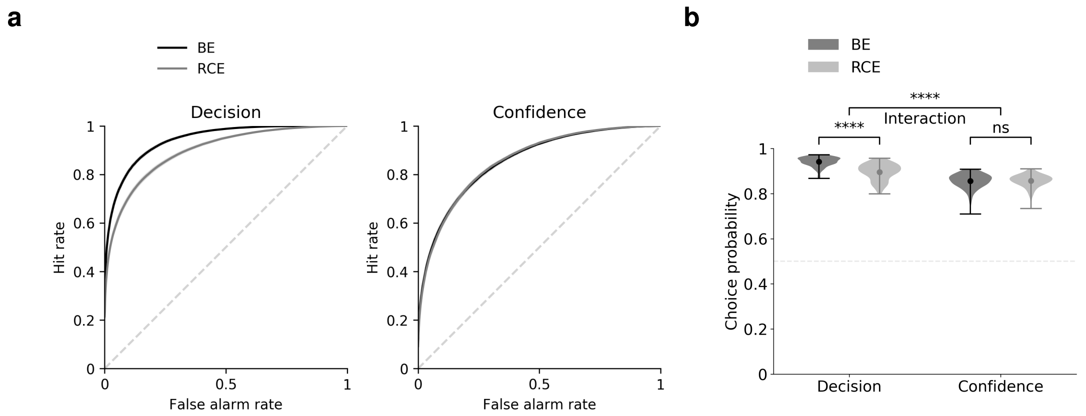

Figure S13: **Sensitivity to neural evidence for vs. against decisions.** Peters et al. [15] performed a neural decoding analysis to test the predictions of the BE and RCE models. Participants performed a face/house discrimination task, and decoders were trained to use cortical electrophysiological signals to predict the stimulus category (face vs. house). This provided an estimate of the independent neural evidence in favor of faces and houses, which could be used to compute, on a trial-by-trial basis, either the balance of evidence or the response-congruent evidence alone. ROC and choice probability (area under the ROC curve) analyses were performed to determine to what extent these neural evidence measures could predict decisions and confidence ratings. Our neural network model displayed the same pattern as observed by Peters et al. (**(a)** ROC analyses, **(b)** choice probability analyses) when decoding from all layers. Decisions were predicted significantly better by the BE rule than the RCE rule (two-sided paired t-test for decision choice probability, BE vs. RCE: $p = 9.4 \times 10^{-45}$). Confidence ratings were predicted equally well by both rules (two-sided paired t-test for confidence choice probability, BE vs. RCE: $p = 0.6$; interaction: $p = 6.9 \times 10^{-38}$). Panel (a) shows average ROC over 100 trained networks $\pm$ the standard error of the mean. Panel (b) shows probability density over 100 trained networks, with mean choice probability in each condition represented by circular markers, and maxima/minima represented by the upper/lower line; ns indicates $p > 0.05$, **** indicates $p < 0.0001$. Source data are provided as a Source Data file.

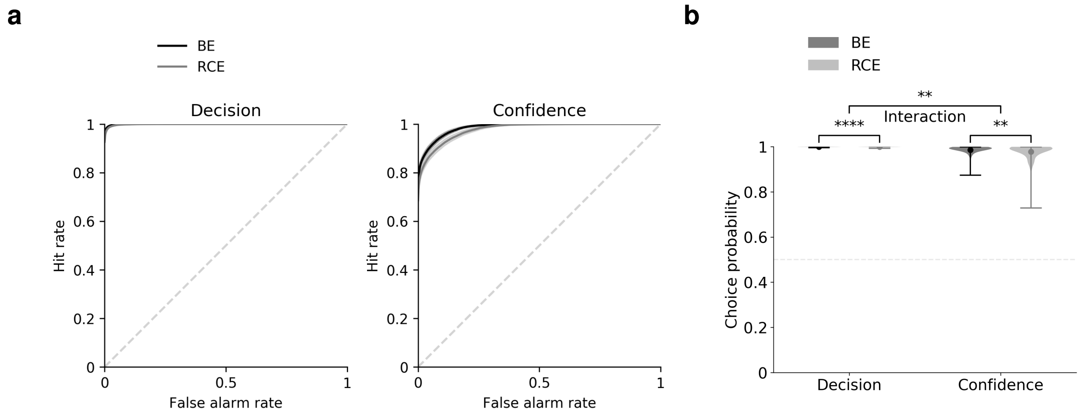

Figure S14: **Decoding analysis in penultimate layer yields results consistent with presence of common decision variable.** The decoding analysis described in Figure S13, originally applied to a readout of the entire network, was applied here to the penultimate layer (output of the encoder f) only. The original analysis indicated that decisions were better predicted by the balance of neural evidence, whereas confidence was equally well predicted by the balance of evidence and the response-congruent evidence alone. In contrast to those results, when the decoding analysis was applied to the penultimate layer only, the results were consistent with the presence of a common decision variable. Panels **(a)** and **(b)** show that both decisions and confidence were better predicted by the BE than the RCE rules, inconsistent with the results of the original decoding analysis (two-sided paired t-tests; decision choice probability, BE vs. RCE: $p = 2.5 \times 10^{15}$; confidence choice probability, BE vs. RCE: $p = 0.0034$; interaction: $p = 0.0052$). Furthermore, to the extent that an interaction was present, it was in the opposite direction of the interaction in the original results (the BE vs. RCE difference is greater for confidence than for decisions). However, we caution that these results are subject to a ceiling effect – decoding accuracy is very high for all conditions, due to the fact that the layer in question already represents the to-be-decoded variables in a linearly separable manner that can be read out by the subsequent output layers. To address this confound, we also performed a version of the decoding analysis in which additional noise was added to the activations of the penultimate layer before being passed to the decoder (Figure S15). Panel (a) shows average ROC over 100 trained networks ± the standard error of the mean. Panel (b) shows probability density over 100 trained networks, with mean choice probability in each condition represented by circular markers, and maxima/minima represented by the upper/lower line; ** indicates $p < 0.01$, **** indicates $p < 0.0001$. Source data are provided as a Source Data file.

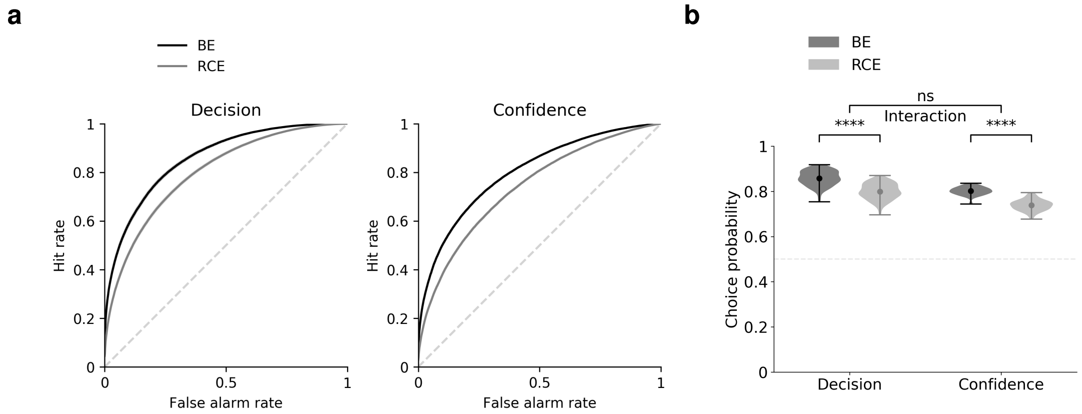

Figure S15: **Decoding analysis in penultimate layer yields results consistent with presence of common decision variable (continued from Figure S14).** To address the ceiling effect in Figure S14, we also performed a version of the decoding analysis in which additional noise (sampled from a Gaussian distribution with a variance of 2) was added to the activations of the penultimate layer before being passed to the decoder. This allowed us both to eliminate the ceiling effect, and to simulate the instrument noise present in real neural decoding analyses. Under these conditions, both decisions and confidence were better predicted by the BE than the RCE rules (two-sided paired t-tests; decision choice probability, BE vs. RCE: $p = 2.4 \times 10^{112}$; confidence choice probability, BE vs. RCE: $p = 4.2 \times 10^{54}$), and there was no interaction between decision rule and dependent variable (two-sided paired t-test: $p = 0.058$), consistent with the presence of a common decision variable. Panel (a) shows average ROC over 100 trained networks $\pm$ the standard error of the mean. Panel (b) shows probability density over 100 trained networks, with mean choice probability in each condition represented by circular markers, and maxima/minima represented by the upper/lower line; ns indicates $p > 0.05$, **** indicates $p < 0.0001$. Source data are provided as a Source Data file.

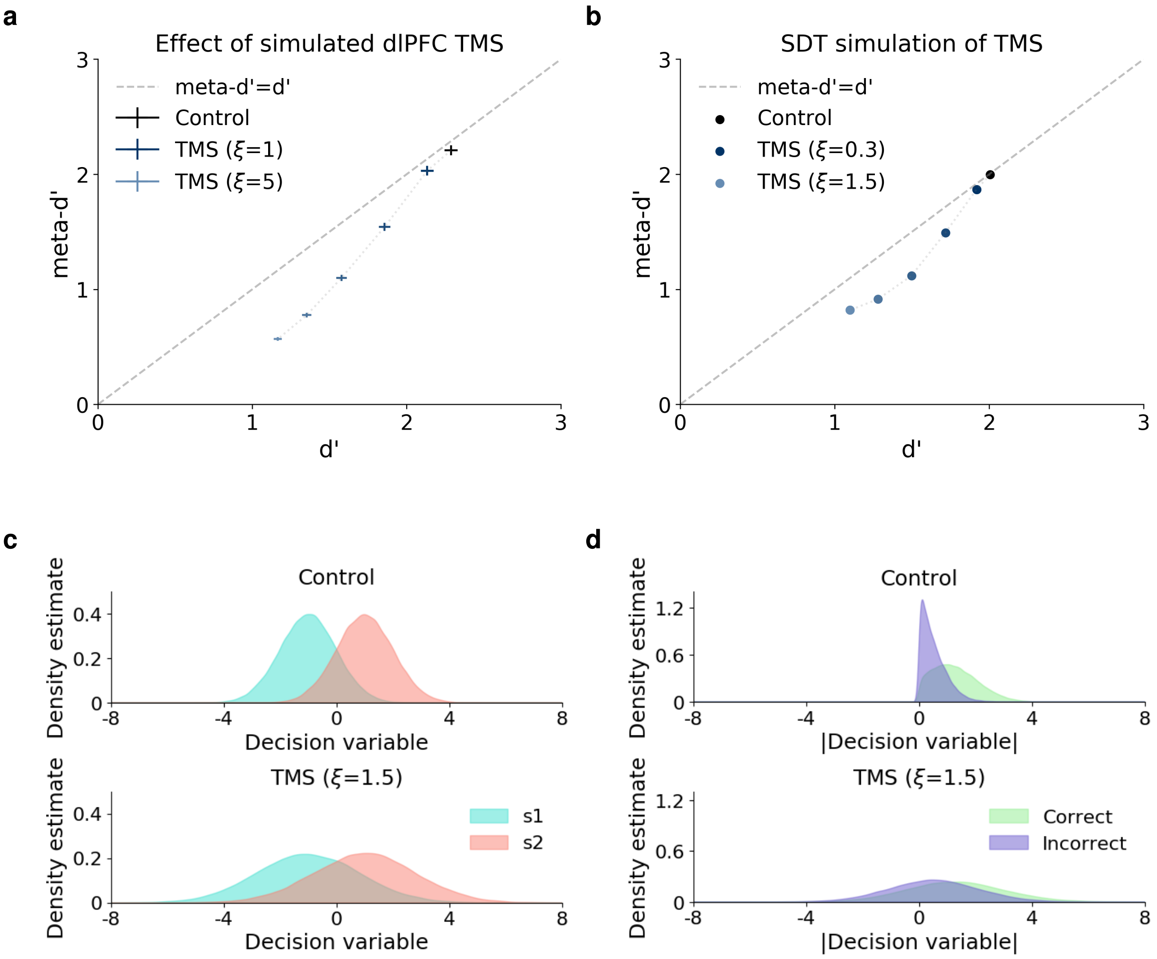

Figure S16: **Simulated TMS to dlPFC impairs type-2 sensitivity more than type-1 sensitivity.** TMS to dlPFC was simulated by adding Gaussian noise with variance ξ to the activations of the penultimate layer. **(a)** This had a greater impact on type-2 sensitivity, as measured by meta-d', than it did on type-1 sensitivity, as measured by d'. Results reflect average over 100 trained networks $\pm$ the standard error of the mean. To better understand this, we performed a simple unidimensional signal detection theory (SDT) simulation. In this simulation, decision-making and confidence were modeled using a single decision variable. On each trial, the decision variable was sampled either from the distribution for s1 (with a mean of -1) or for s2 (with a mean of 1), both of which had a variance of 1. Decisions were based on the sign of the decision variable, and confidence was based on the absolute value of the decision variable. TMS to the penultimate layer was modeled by adding Gaussian noise both to the decision variable (for simulating decisions and calculating d'), and to the absolute value of the decision variable (for simulating confidence and calculating meta-d'). The addition of noise after the rectification of the decision variable distinguishes this simulation from standard detection-theoretic models, and was motivated by the fact that the penultimate layer contains neurons that represent both the decision variable (PC1) and the rectified decision variable (PC2). **(b)** This simple model successfully captured the effect of greater impairment to type-2 vs. type-1 sensitivity following simulated TMS via the injection of noise to this layer. This may explain the selective impairment of meta-d' observed following TMS to dlPFC [24]. This effect can be intuitively understood by observing the impact of noise on **(c)** the distribution of the decision variable for s1 and s2 trials vs. **(d)** the distribution of the rectified decision variable for correct and incorrect trials. The same level of noise results in greater overlap for correct and incorrect distributions than it does for s1 and s2 distributions. Source data are provided as a Source Data file.

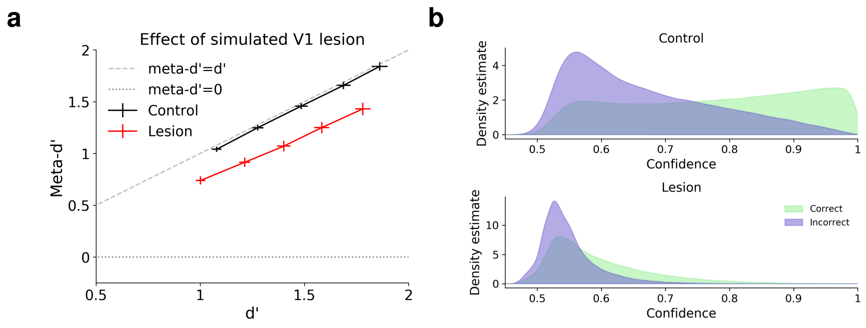

Figure S17: **Simulating less severe metacognitive impairment following V1 lesion.** The exact extent of the metacognitive impairment in blindsight is unclear. Some data suggests that, although meta-d' is significantly lower than would be expected based on d', it may still be above zero in some blindsight patients [26]. Our model can account for this pattern as well, by using a larger scaling factor (corresponding to the relative strength of visual inputs from intact subcortical regions). Here, we present results with a scaling factor of 0.5. **(a)** Meta-d' is reduced, but still significantly above zero, following lesion. **(b)** Confidence distributions for correct and incorrect trials are not completely overlapping following lesion. Source data are provided as a Source Data file.

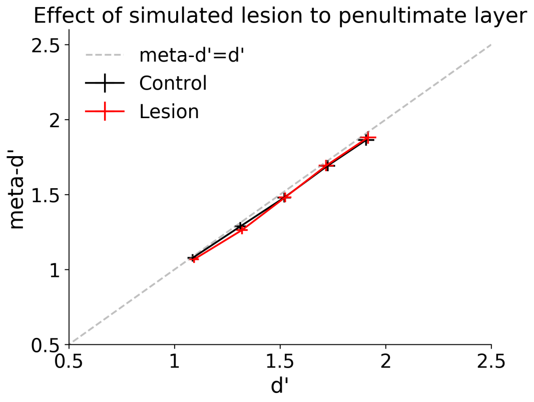

Figure S18: **Simulated lesion to penultimate layer has no effect on type-2 sensitivity.** A lesion to the penultimate layer of the network was simulated by scaling the activations in this layer by a factor of 0.01 (as was done in the first layer of the network to simulate blindsight). This had no impact on either type-1 sensitivity (d') or type-2 sensitivity (meta-d'). Source data are provided as a Source Data file.
