# Supplementary material for: Natural statistics support a rational account of confidence biases
Source: Nat Commun. 2023 Jul 6;14:3992. doi: 10.1038/s41467-023-39737-2 (PMC10326055; doi:10.1038/s41467-023-39737-2)
Supplement: Supplementary file 3 — Reporting Summary [file 41467_2023_39737_MOESM3_ESM.pdf]

nature portfolio

Corresponding author(s): Taylor Webb

Last updated by author(s): Apr 4, 2023

Reporting Summary

Nature Portfolio wishes to improve the reproducibility of the work that we publish. This form provides structure for consistency and transparency in reporting. For further information on Nature Portfolio policies, see our Editorial Policies and the Editorial Policy Checklist.

Statistics

For all statistical analyses, confirm that the following items are present in the figure legend, table legend, main text, or Methods section.

n/a	Confirmed	
☐	☒	The exact sample size (*n*) for each experimental group/condition, given as a discrete number and unit of measurement
☐	☒	A statement on whether measurements were taken from distinct samples or whether the same sample was measured repeatedly
☐	☒	The statistical test(s) used AND whether they are one- or two-sided<br>*Only common tests should be described solely by name; describe more complex techniques in the Methods section.*
☐	☒	A description of all covariates tested
☐	☒	A description of any assumptions or corrections, such as tests of normality and adjustment for multiple comparisons
☐	☒	A full description of the statistical parameters including central tendency (e.g. means) or other basic estimates (e.g. regression coefficient) AND variation (e.g. standard deviation) or associated estimates of uncertainty (e.g. confidence intervals)
☐	☒	For null hypothesis testing, the test statistic (e.g. *F*, *t*, *r*) with confidence intervals, effect sizes, degrees of freedom and *P* value noted<br>*Give P values as exact values whenever suitable.*
☒	☐	For Bayesian analysis, information on the choice of priors and Markov chain Monte Carlo settings
☒	☐	For hierarchical and complex designs, identification of the appropriate level for tests and full reporting of outcomes
☒	☐	Estimates of effect sizes (e.g. Cohen's *d*, Pearson's *r*), indicating how they were calculated

Our web collection on statistics for biologists contains articles on many of the points above.

Software and code

Policy information about availability of computer code

Data collection

Code for all simulations and analyses is available at: https://github.com/taylorwwebb/performance_optimized_NN_confidence
The following tools/packages were used:
Python 3
PyTorch
NumPy
SciPy
scikit-learn
Matplotlib
colorlog
PIL

Data analysis	Code for all simulations and analyses is available at: https://github.com/taylorwwebb/performance_optimized_NN_confidence<br>The following tools/packages were used:<br>Python 3<br>PyTorch<br>NumPy<br>SciPy<br>scikit-learn<br>Matplotlib<br>colorlog<br>PIL

For manuscripts utilizing custom algorithms or software that are central to the research but not yet described in published literature, software must be made available to editors and reviewers. We strongly encourage code deposition in a community repository (e.g. GitHub). See the Nature Portfolio guidelines for submitting code & software for further information.

Data

Policy information about availability of data

All manuscripts must include a data availability statement. This statement should provide the following information, where applicable:

- Accession codes, unique identifiers, or web links for publicly available datasets
- A description of any restrictions on data availability
- For clinical datasets or third party data, please ensure that the statement adheres to our policy

All data are provided in a Source Data file.

Human research participants

Policy information about studies involving human research participants and Sex and Gender in Research.

Reporting on sex and gender	All results reflect computer simulations. These simulations model qualitative effects reported in previous behavioral studies.
Population characteristics	There are no population characteristics, as the results are derived only from computer simulations with neural network and Bayesian ideal observer models.
Recruitment	There was no recruitment procedure, as the results are derived only from computer simulations with neural network and Bayesian ideal observer models.
Ethics oversight	There was no need for ethics oversight, as the results are derived only from computer simulations with neural network and Bayesian ideal observer models.

Note that full information on the approval of the study protocol must also be provided in the manuscript.

Field-specific reporting

Please select the one below that is the best fit for your research. If you are not sure, read the appropriate sections before making your selection.

☐ Life sciences ☒ Behavioural & social sciences ☐ Ecological, evolutionary & environmental sciences

For a reference copy of the document with all sections, see nature.com/documents/nr-reporting-summary-flat.pdf

Behavioural & social sciences study design

All studies must disclose on these points even when the disclosure is negative.

Study description	Computer simulations modeling qualitative phenomena described in previous behavioral studies. These phenomena involve decision making behavior, confidence ratings, and neural measures.
Research sample	The study sample consisted of artificial neural networks.
Sampling strategy	No statistical methods were used to determine sample size.
Data collection	Data involves behavioral measures and internal states from artificial neural networks. Researchers were not blinded to the experimental conditions or study hypotheses.
Timing	Data was collected between July 2020 and November 2022.

Data exclusions	No data were excluded from analysis.
Non-participation	There were no participants. The study was performed on artificial neural networks.
Randomization	All artificial neural networks were randomly initialized at the beginning of training.

Reporting for specific materials, systems and methods

We require information from authors about some types of materials, experimental systems and methods used in many studies. Here, indicate whether each material, system or method listed is relevant to your study. If you are not sure if a list item applies to your research, read the appropriate section before selecting a response.

Materials & experimental systems

n/a	Involved in the study	
☒	☐	Antibodies
☒	☐	Eukaryotic cell lines
☒	☐	Palaeontology and archaeology
☒	☐	Animals and other organisms
☒	☐	Clinical data
☒	☐	Dual use research of concern

Methods

n/a	Involved in the study	
☒	☐	ChIP-seq
☒	☐	Flow cytometry
☒	☐	MRI-based neuroimaging
